# Effectiveness of *Salvadora persica* toothbrush and *Salvadora persica* chewing stick in plaque and gingivitis control: a randomized control trial

**DOI:** 10.1186/s12906-023-04295-z

**Published:** 2023-12-14

**Authors:** Nurul Fatin Azizan, Nurulhuda Mohd, Nik Madihah Nik Azis, Badiah Baharin

**Affiliations:** Department of Restorative Dentistry, Faculty of Dentistry, Universiti Kebangsaan Malaysia, Jalan Raja Muda Abdul Aziz, 50300 Kuala Lumpur, Malaysia

**Keywords:** Biofilm, Inflammation, Natural product, Oral hygiene, *Salvadora persica*

## Abstract

**Background:**

The values of plant-based products have taken on an expanding relevance in dentistry*. Salvadora persica* chewing stick (miswak) has been practiced for centuries and is recommended by the World Health Organization as a customary oral hygiene tool. The therapeutic effects of *S. persica* chewing stick are contributed by its mechanical cleansing action, active chemicals released, or the combination of these two actions. However, the *S. persica* chewing stick in its natural form can be difficult to maneuver in certain parts of the mouth. This concern has inspired the innovation of the *S. persica* toothbrush that is designed to merge the ease of use of a toothbrush with the beneficial natural properties of *S. persica* preserved in its bristle. The present study aimed to compare the clinical effectiveness between *S. persica* toothbrush, *S. persica* chewing stick and the standard toothbrush in plaque and gingivitis control.

**Methods:**

In this single-blinded and parallel randomized controlled trial, 78 participants were randomly divided into three groups to either use (i) *S. persica* toothbrush (MTB); (ii) *S. persica* chewing stick (MCS); or (iii) standard toothbrush (STB) in a standardized manner for three weeks. Plaque Index (PI) and Periodontal Inflamed Surface Area (PISA) values, measuring plaque levels and severity of gingivitis, respectively, were evaluated at baseline, one- and three-week post-interventions.

**Results:**

The MCS group showed a significant improvement in the mean PISA values of the anterior teeth compared to the MTB and STB groups (MCS: from 16.35 ± 10.03 to 3.41 ± 1.14; MTB: from 25.20 ± 14.01 to 3.57 ± 1.19; STB: from 26.54 ± 8.64 to 6.17 ± 0.86; *p* < .050). All three groups reported significant improvements (*p* < .001) in the plaque levels and the severity of gingivitis from baseline to three weeks after the intervention.

**Conclusions:**

Following correct techniques, *S. persica* toothbrush and chewing sticks are as effective as the standard toothbrush in plaque control and gingival health, which represent the reputed anti-plaque and anti-gingivitis properties of *S. persica*.

**Trial registration:**

This clinical trial was prospectively registered in ClinicalTrials.gov with registration NCT04650685 (25/11/2020).

## Introduction

Dental biofilm is a highly organized biological environment that consists of complex microbial community [1]. The bacteria form sophisticated networks hence are largely protected from host defenses and resistant to the action of chemotherapeutic agents [2]. Dental biofilm is widely acknowledged as the fundamental driver of dental caries formation and the development of periodontal diseases, which constitute gingivitis and periodontitis [3]. Gingivitis, characterized by bleeding gums, is an inflammatory condition of the gingiva [4]. Dental biofilm contributes to the progression of gingivitis to periodontitis, which represents a more severe stage of periodontal disease [5]. Therefore, gingivitis control is a crucial first line of defense against periodontitis [6].

Dental caries is caused by a complex interplay between acid-forming biofilm microorganisms in the presence of sugar [7]. The metabolism of sugar by dental biofilm bacteria also leads to the formation of short-chain carboxylic acids, which in turn may contribute to gingival inflammation [8]. Moreover, the overwhelming prevalence of dental caries and periodontal disease is strongly linked to behavior, particularly with regards to unsatisfactory oral hygiene and a high dietary intake of carbohydrates [7]. Thus, while sugar restriction has been associated with reduced gingival inflammation and caries development [8], interventional techniques and armamentarium are indispensable to help curb the disease progression and further affects individual’s quality of life [9]. This preventive strategy involves physically disrupting dental plaque biofilms and reducing the bacterial bioburden through effective mechanical oral hygiene measures, such as toothbrushing [10].

Nowadays, the global population thrives in its interest towards using natural products as oral hygiene care apparatus*.* This growing interest has led to a demand for safe, effective, and economical alternative prevention methods on a global scale [11]. Researchers worldwide developed a multilevel strategy concentrating on natural products as an alternative option for prevention. This initiative is in response to heightened efforts to improve oral health education and lessen the economic burden associated with oral diseases [12]. *Salvadora persica* chewing stick, often known as miswak, is used natural products due to its versatility in function and benefits for oral hygiene care [13]. Prepared from trees of the family *Salvadoraceae* [12], these chewing are made fromstems and roots with thick-walled fibres that make them spongy in texture [13], which swell and soft when submerged in the water [14]. As the name implies, these chewing sticks are readily chewed and crushed between the teeth, where the fibres on one end of the sticks fraying to resemble the bristle of a toothbrush [15].

*S. persica* chewing sticks have a long tradition of use for being natural, affordable, and readily accessible [16]. The preference of using these sticks is also linked to the cultural and religious influences [17]. The World Health Organization encourages using *S. persica* chewing sticks as an effective oral hygiene tool in areas where it is customary [18]. Studies showed that *S. persica* chewing stick considerably contributes to promoting oral hygiene [19], especially in countries with financial restraints or limited access to oral healthcare services for the general population [20].

 The vast diversity of bioactive natural compounds in *S. persica* chewing sticks [21], particularly benzyl isothiocyanate (BITC), contribute to their antimicrobial activity [22–24]. An in vitro examination of *S. persica* chewing sticks reported BITC, the main compound in *S. persica* essential oils, exerted potent antimicrobial actions against the exceptionally destructive periodontopathic pathogens such as *Porphyromonas gingivalis* and *Aggregatibacter actinomycetemcomitans* [1]. In vitro studies also showed that BITC exhibit synergistic anti-plaque activity against primary colonizers of dental plaque [25, 26], inhibiting both plaque formation [27, 28] and demonstrating bacteriostatic properties against cariogenic bacteria such as *Streptococcus mutans* by retarding their acid production [29, 30].

A recent systematic review reported that the *S. persica* chewing sticks are as effective as standard toothbrushes in mechanically removing plaque build-up [12]. Additionally, they were reported to possess superior anti-gingivitis properties compared to the standard toothbrush [12]. Silica found in the *S. persica* extracts enhance the mechanical action of plaque removal, while sodium bicarbonate has a mild abrasive effect to act as a potential whitening agent [31, 32]. Tannins and resins serve as anti-gingivitis agents, exerting an astringent effect on the mucous membrane and reducing clinically detectable gingival inflammation, thereby promoting periodontal health [22, 33, 34]. Clinical studies demonstrated that *S. persica* chewing sticks users yielded significantly lower mean plaque accumulation [35] and gingival bleeding scores [2] compared to standard toothbrush users. Thus, these combined effects of mechanical cleaning action and various bioactive constituents of *S. persica* chewing sticks safeguard against plaque accumulation and clinically detectable gingivitis [12].

Nevertheless, the difficulty in maneuvering *S. persica* chewing stick in practice, the practicality of storing the unused portion of the chewing stick, or the general unattractiveness of the raw and unprocessed chewing stick itself hinder the use [12, 19]. Understanding these concerns, the *S. persica* toothbrush is designed to merge the everyday practicality of a standard toothbrush, with bristles made from *S. persica* powder and natural silica. The added component of natural silica enhances the mechanical action of this toothbrush in plaque removal. Introducing this innovative *S. persica* toothbrush into the global market may offer a potential avenue for expanding the benefits of natural resources in routine oral hygiene [14]. However, it is emphasized that scientific evidence is essential to validate the therapeutic claims of this public-appealing product to safeguard its safety and efficacy in the context of oral health. To date, no study is available to test the efficacy of this *S. persica* toothbrush compared to the standard toothbrush. Therefore, the primary aim of this study was to determine the effectiveness of *Salvadora persica* oral hygiene tools and standard toothbrush in improving plaque control and gingival health. The secondary aim was to compare the oral health status on gingivitis and plaque control between *Salvadora persica* toothbrush and *Salvadora persica* chewing stick usage in a standardized manner.

## Materials and methods 

### Ethical aspects

The research protocol was approved by the Ethical Committee of Universiti Kebangsaan Malaysia, Kuala Lumpur, Malaysia (JEP-2020–620). This study was registered with ClinicalTrials.gov (ID: NCT04650685, 25/11/2020) and conducted in accordance with the Declaration of Helsinki [36]. The study is presented in accordance with the Consolidated Standards of Reporting Trials (CONSORT) guidelines [37].

### Study design

**Fig. 1:**
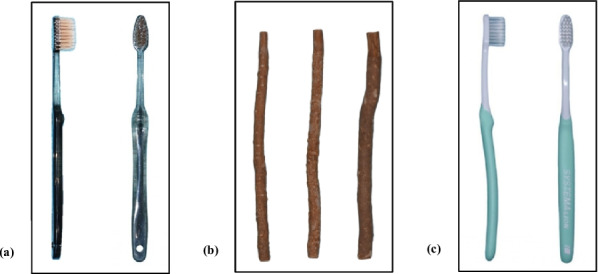
The oral hygiene tools used in the study. **a** The *Salvadora persica* toothbrush Al-Abyad Miswak® (Insight Prestige Sendirian Berhad, Kuala Lumpur, Malaysia) (**b**) The *Salvadora persica* chewing sticks Al Khair Miswak® (Al Khair, Karachi, Pakistan) (**c**) The standard toothbrush Compact Systema.® (Lion, Tokyo, Japan)

This single-center study was a 21-day experimental trial with a randomized, parallel, single-blind design that consisted of three arms: Group 1 (MTB): *Salvadora persica* toothbrush, Group 2 (MCS): *Salvadora persica* chewing stick, and Group 3 (STB): Standard toothbrush and toothpaste (control). The oral hygiene tools used based on the groups are presented in Fig. 1.

### Study population

Convenience sampling was used in this study involving non-dental students and staff of Universiti Kebangsaan Malaysia, Kuala Lumpur Campus. The study was conducted in Faculty of Dentistry, Universiti Kebangsaan Malaysia from July 2021 until February 2022. The PICO (*P* = Patient population; I = Intervention of interest; C = Comparative intervention; O = Outcome) question for this study was, ‘In adult patients with gingivitis (P), what are the short-term effects of using *S. persica* oral hygiene tools (toothbrush or chewing stick (I) versus the standard toothbrush (C) on dental plaque and gingival inflammation (O)?’. Consent was obtained prior to the subject recruitment where the protocol of the study including the benefits and risks were explained in detail.

### Inclusion criteria

Individuals who:

- were systemically healthy.
- had ≥ 20 teeth.
- had Basic Periodontal Examination score 0, 1 and 2 only with no periodontal pockets more than 5.5 mm.
- had never smoked cigarettes or other tobacco products.

### Exclusion criteria

Individuals who:

- were habitual users of miswak chewing sticks or have experienced in using them.
- had orthodontic appliances.
- had grossly carious teeth, gross overhanging restorations, severe malpositioned teeth and/or gingival recession, had crowns placed, and wore partial dentures.
- had current or previous history of periodontal treatment including root surface debridement/periodontal surgery.
- had poor manual dexterity.
- were pregnant or were lactating mothers.
- had taken antibiotics in the previous 3 months.

### Sample size

The sample size was estimated using a mean plaque score difference of 0.19, a pooled standard deviation of 0.23, a significance level of 5% and a power of 80% [2]. A sample size of 24 subjects in each arm was required for a parallel design. Considering the 5% drop-out rate of maximum two participant drop-out from each group, a minimum of 78 eligible participants were recruited in total.

### Randomization and blinding

Participants were randomized to one of the three groups by a research assistant (NSMZ) using a computerized-generated table. A balanced randomly permuted block of 3 and 6 was used to avoid unequal numbers between the groups. To conceal assignment, an opaque sequentially numbered envelope containing the allocated intervention arm was opened at the baseline visit to allocate the respective participant into one arm of the study. One principal examiner (NFA) was responsible for the clinical evaluations and was blinded to the intervention groups throughout the study. Each participant was identified by a code. Although the blinding of participants was not possible, they were not allowed to disclose the oral hygiene tool they used to the examining dentist.

### Clinical protocol

The clinical protocol was conducted for five weeks, with a total of 4 clinical appointments performed throughout the study. At Visit 1, all participants received professional dental cleaning consisting of scaling and polishing to ensure they received the same standard of care. Participants were instructed to continue their routine oral hygiene habits for the next two weeks. Visit 2, i.e., the baseline period (T0), was then arranged two weeks after. In this visit, proper techniques of using the designated oral hygiene tool were verbally and visually explained using models to each participant by another trained dentist (NIMN). All participants also received pamphlets and videos demonstrating the appropriate techniques of their designated oral hygiene tool. Visit 3 and Visit 4 were arranged at one-week (T1) and three-week (T2), respectively, following the use of the designated oral hygiene tool. Participants were strictly refrained from using other means of cleaning devices, dentifrices, or adjuncts during the study period.

### Outcome variables 

The clinical outcomes were assessed at every visit, measuring:

- Plaque levels; assessed using the Plaque Index (PI) at six sites per tooth after using a disclosing solution of Tri Plaque ID Gel (GC Malaysia, Kuala Lumpur, Malaysia) [38].
- Severity of gingivitis; assessed by the Periodontal inflamed surface area (PISA) at six sites per tooth [39]. Periodontal attachment levels were first measured using a Periodontal Community Probe-University of North Carolina probe (PCP-UNC 15) probe (HuFriedyGroup, Chicago, United States) and the values were transferred to a Microsoft Excel spreadsheet constructed by Hujoel et al. 2001 [40], which is freely available at www.parsprototo.info, to facilitate Periodontal Attachment Surface Area (PESA) and Periodontal Inflamed Surface Area calculations (PISA). PESA quantifies the surface area of the periodontal pocket that also includes the healthy pocket epithelium, without quantifying the surface area of the inflamed pocket epithelium [39]. PISA is the inflamed component of PESA that was clinically affected by bleeding on probing. PISA was represented by bleeding on probing (BOP), which is an important clinical parameter to manifest the severity of gingivitis [6]. It reflected the surface area of bleeding pocket epithelium in square millimeters that quantifies the amount of inflamed periodontal tissue [39].

Prior to the study, the examiner (NFA) was trained and calibrated for accuracy and repeatability using the PI and PISA in five individuals who were not participating in this study. Training and calibration of the examiner were conducted by an experienced periodontist (BB). Inter- and intra-examination Cohen’s Kappa scores showed a percentage of agreement of > 85% in both parameters.

### Standardization of oral hygiene tools

All participants were issued with identical oral hygiene tools according to their assigned groups to standardize the experimental conditions (Fig. 1).

- Group 1 (MTB): A *Salvadora persica* toothbrush (Al-Abyad Miswak®) was identical in size and width to the standard toothbrush, which was used as instructed by the manufacturer.
- Group 2 (MCS): *Salvadora persica* chewing sticks (Al Khair®) were prepared into equal lengths of 20 cm (8 inches long) with a uniform diameter (1.0–1.5 cm) and were sealed in air-tight plastic bags. Three miswak chewing sticks were distributed to each participant (one stick/week).
- Group 3 (STB): Participants in this group received the small-head, soft-bristle Compact Systema® toothbrush and Colgate® Maximum Cavity Protection (Great Regular Flavor) toothpaste.

Participants were instructed to use the designated oral hygiene tool twice daily. Additionally, participants were asked to bring along the designated oral hygiene tools at each visit for the compliance assessment of the oral hygiene methods.

### Standardization of oral hygiene technique protocol

**Fig. 2:**
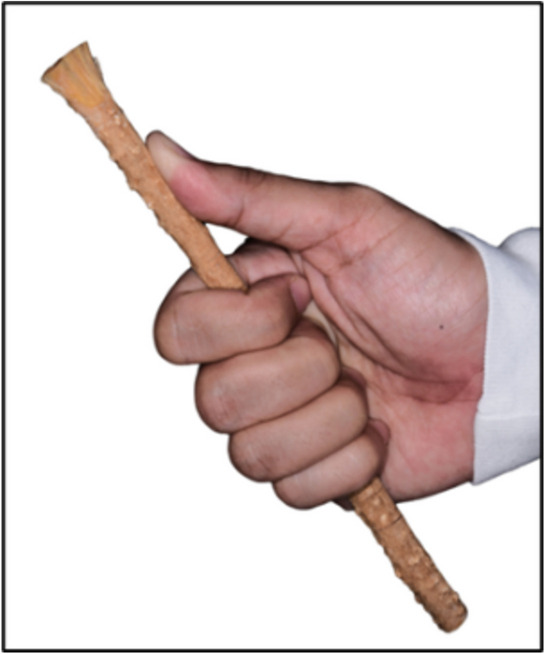
The five-finger grip technique

For the MTB and STB groups, the modified Bass technique was adapted with a minimum two-minute duration to increase plaque removal efficiency [41]. For the MCS group, the five-finger grip technique (Fig. 2) was applied to ensure all tooth surfaces were accessible and cleaned with convenience and controlled movements of the chewing stick [33]. *S. persica* chewing stick should be held perpendicular to the tooth surface and gently moved in up and down motions during cleaning [33]. Gentle motion should also be applied on the gingival margin to massage the gingiva, while avoiding gingival trauma and possible gingival recession [33, 42]. Thus, careful movement during toothbrushing is important to prevent damaging the soft tissues of the oral cavity. The participants were first requested to soak each new *S. persica* chewing stick intended for current use in freshwater 24 h before use to facilitate softening its natural fibers [43].This would facilitate the fiber crushing and release of its chemical constituents [43], hence highly unlikely to traumatize the gingiva while brushing [14].

**Fig. 3:**
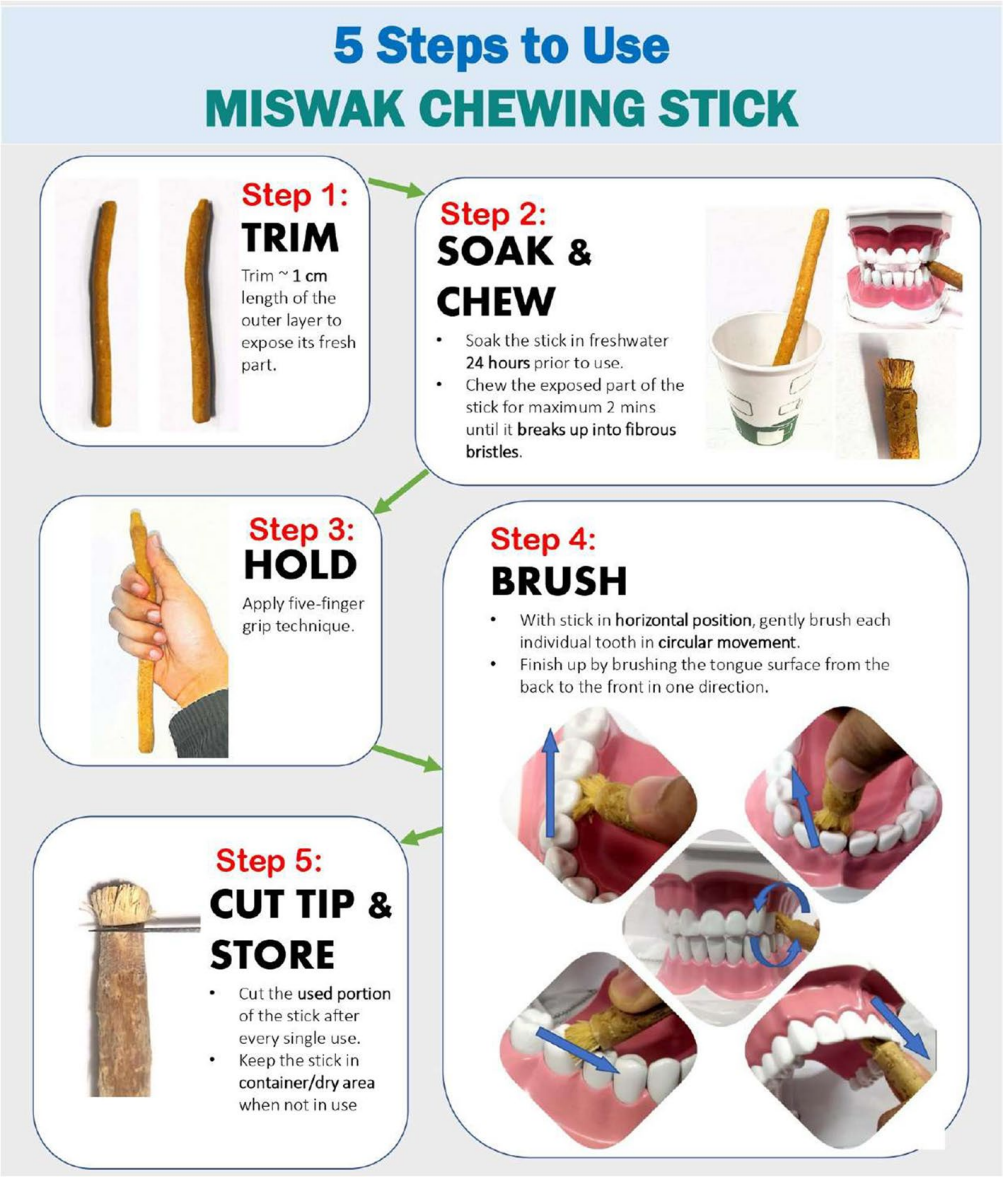
The steps of using the *Salvadora persica* chewing sticks

Participants were reminded to cut the used portion of the *S. persica* chewing stick daily. Participants of the MCS group were not selected among the habitual *S. persica* chewing stick users hence were expected to spend a longer time familiarizing themselves with the appropriate brushing techniques using the chewing stick [44]. Thus, a five-minute duration was advocated for the MCS group [19]. The steps to use the *S. persica* chewing sticks were illustrated as shown in Fig. 3.

### Data analysis

Study data were entered into Statistical Package for Social Sciences version 25 (SPSS Inc., Chicago, IL, USA) using anonymous patient codes. The baseline characteristics and the clinical outcomes were reported as mean with standard deviation for the continuous variables (age, Plaque Index, and PISA values). These variables had normal distributions i.e., tested for normality using the Shapiro–Wilk test, hence were analyzed using mixed model analysis of variance to enhance the capabilities of greater statistical power. The categorical variables of baseline characteristics were presented as frequencies and percentages, which were tested using Fisher’s exact test. Statistical significance was set at the 95% confidence level (α = 0.05), in which a *p*-value of < 0.05 was considered statistically significant.

## Results

### Study population

**Fig. 4:**
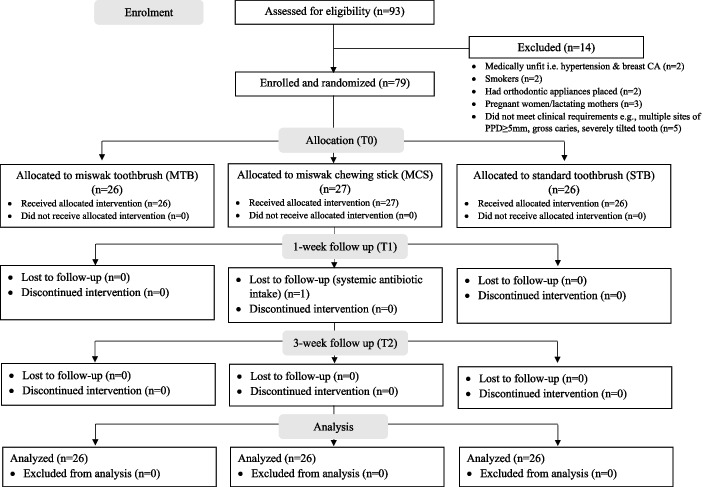
Study flowchart following CONSORT guidelines for clinical trials

Seventy-nine participants who fulfilled the inclusion and exclusion criteria were enrolled. However, only 78 participants remained, as one participant had to drop out after taking systemic antibiotic course following a persistent ear infection. All 78 participants completed the study, as depicted in the study flowchart in Fig. 4.

### Demographic background and baseline data

A summary of participant demographics and baseline periodontal parameters are presented in Table 1. Study participants ranged from 19 to 42 years old, with a mean age of 25.4 years. No significant differences in age, gender, ethnicity, and/or background education levels were noted between groups at baseline. A low baseline mean Plaque Index was seen across all groups, with no significant difference between the groups [0.18, 0.13, and 0.12, respectively (*p* = 0.182)]. No significant difference was recorded when PISA values (severity of gingivitis) were compared between groups [77.82, 69.92, and 69.42, respectively (*p* = 0.839)].

**Table 1:** Demographic data and baseline periodontal parameters of the study population

Variables	Group 1 (MTB) (*n* = 26)	Group 2 (MCS) (*n* = 26)	Group 3 (STB) (*n* = 26)	*p*-value
Age				
Mean (± SD); years	25.6 (± 6.7)	26.2 (± 7.8)	24.5 (± 5.5)	.638^a^
Range; years	19–40	19–42	19–41	
Gender				
Male; n (%)	4 (15.4%)	4 (15.4%)	6 (23.1%)	.706^b^
Female; n (%)	22 (84.6%)	22 (84.6%)	20 (76.9%)	
Ethnicity				
Malay; n (%)	23 (88.5%)	23 (88.5%)	20 (76.9%)	.939^b^
Chinese; n (%)	1 (3.8%)	1 (3.8%)	2 (7.7%)	
Indian; n (%)	1 (3.8%)	1 (3.8%)	2 (7.7%)	
Others; n (%)	1 (3.8%)	1 (3.8%)	2 (7.7%)	
Education level				
Certificate level; n (%)	3 (11.5%)	2 (7.7%)	1 (3.8%)	.348^b^
Diploma level; n (%)	0 (0.0%)	4 (15.4%)	1 (3.8%)	
Degree level; n (%)	20 (76.9%)	17 (65.4%)	20 (76.9%)	
Master/PhD; n (%)	3 (11.5%)	3 (11.5%)	4 (15.4%)	
Plaque Index; Mean ± SD	0.18 ± 0.08	0.13 ± 0.06	0.12 ± 0.05	.182^a^
PISA; Mean ± SD	77.82 ± 37.46	69.92 ± 34.27	69.42 ± 29.74	.839^a^

^a^one-way ANOVA

^b^Fisher’s exact test

### Clinical outcomes

From baseline to three-week post-intervention, the mean differences in the plaque level across the groups were 0.09, 0.03, and 0.04, respectively. The mean differences in the PISA values in the three groups were 59.65, 51.49, and 52.25, respectively. The intra-group analysis for PI and PISA showed statistically significant improvement (*p* < 0.001) in the mean differences of plaque levels and the severity of gingivitis. When different types of oral hygiene tools were compared, no significant difference (*p* > 0.05) was recorded.

Table 2 shows the comparative effects of plaque level and severity of gingivitis on different tooth positions (anterior and posterior teeth), while Table 3 shows the comparison of these outcomes on different tooth surfaces (buccal and palatal/lingual). The effects of both tooth positions and tooth surfaces show similar patterns in the reduction of plaque levels and severity of gingivitis of all groups from baseline (T0) to three weeks after the intervention (T2). Generally, PI and PISA values for anterior teeth were lower than the posterior teeth in all visits across the groups (Table 2), similar to that of buccal surface when compared to the palatal/lingual surfaces in all groups (Table 3).

**Table 2:** The effects of plaque level and severity of gingivitis using *S. persica*-based oral hygiene tools and standard toothbrush on different tooth positions

Variables	Timepoint	Groups						*p*-value*^b^** (anterior teeth)**	*p*-value*^b^** (posterior teeth)**
		**MTB**		**MCS**		**STB**			
		Anterior	Posterior	Anterior	Posterior	Anterior	Posterior		
Plaque Index (Mean ± SD)	T0	0.07 ± .02	0.11 ± .06	0.04 ± .02	0.09 ± .04	0.05 ± .03	0.08 ± .04	MTB vs MCS: 0.376 MTB vs STB: 0.819 MCS vs STB: 0.525	MTB vs MCS: 0.551 MTB vs STB: 0.425 MCS vs STB: 0.768
	T1	0.07 ± .03	0.10 ± .05	0.03 ± .02	0.09 ± .04	0.04 ± .02	0.07 ± .03		
	T2	0.03 ± .02	0.07 ± .03	0.02 ± .01	0.08 ± .03	0.03 ± .02	0.06 ± .03		
	Mean difference^▲^	0.04	0.04	0.02	0.01	0.02	0.02		
	*p*-value*^a^	0.472		0.361		0.618			
Periodontal Inflamed Surface Area (Mean ± SD)	T0	25.20 ± 14.01	53.59 ± 39.77	16.35 ± 10.03	53.86 ± 40.41	26.54 ± 8.64	42.88 ± 43.07	MTB vs MCS: 0.046 MTB vs STB: 0.577 MCS vs STB: 0.041	MTB vs MCS: 0.262 MTB vs STB: 0.794 MCS vs STB: 0.358
	T1	19.81 ± 7.01	28.11 ± 25.33	9.09 ± 4.49	40.89 ± 30.92	7.02 ± 2.82	33.62 ± 20.14		
	T2	3.57 ± 1.19	14.56 ± 9.18	3.41 ± 1.14	15.01 ± 10.61	6.17 ± 0.86	10.96 ± 9.61		
	Mean difference^▲^	21.63	39.03	12.94	38.59	20.37	31.92		
	*p*-value*^a^	0.223		0.037		0.594			

^▲^T0-T2

^*^Mixed Model ANOVA

^a^*p*-value within groups

^b^*p*-value between groups

**Table 3:** The effects of plaque level and severity of gingivitis using *S. persica*-based oral hygiene tools and standard toothbrush on different tooth surfaces

Variables	Timepoint	Groups						*p*-value*^b^** (buccal surface)**	*p*-value*^b^** (palatal / lingual surface)**
		**MTB**		**MCS**		**STB**			
		Buccal	Palatal / Lingual	Buccal	Palatal / Lingual	Buccal	Palatal / Lingual		
Plaque Index (Mean ± SD)	T0	0.04 ± .03	0.14 ± .08	0.03 ± .02	0.10 ± .06	0.04 ± .03	0.09 ± .06	MTB vs MCS: 0.824 MTB vs STB: 0.796 MCS vs STB: 0.885	MTB vs MCS: 0.244 MTB vs STB: 0.481 MCS vs STB: 0.633
	T1	0.03 ± .02	0.13 ± .08	0.02 ± .02	0.10 ± .05	0.02 ± .02	0.09 ± .05		
	T2	0.01 ± .02	0.08 ± .05	0.01 ± .01	0.09 ± .03	0.02 ± .02	0.06 ± .05		
	Mean difference^▲^	0.03	0.06	0.02	0.01	0.02	0.03		
	*p*-value*^a^	0.504		0.862		0.781			
Periodontal Inflamed Surface Area (Mean ± SD)	T0	18.97 ± 13.91	59.76 ± 39.92	14.92 ± 10.04	55.13 ± 39.36	19.80 ± 13.79	49.54 ± 26.54	MTB vs MCS: 0.327 MTB vs STB: 0.719 MCS vs STB: 0.542	MTB vs MCS: 0.486 MTB vs STB: 0.221 MCS vs STB: 0.538
	T1	11.49 ± 6.17	36.29 ± 23.85	11.04 ± 6.90	38.72 ± 25.22	7.28 ± 3.88	33.19 ± 21.59		
	T2	3.89 ± 1.28	14.24 ± 9.65	3.29 ± 0.94	15.10 ± 9.83	5.62 ± 2.46	11.48 ± 6.11		
	Mean difference^▲^	15.08	45.52	11.63	40.03	14.18	38.06		
	*p*-value*^a^	0.439		0.046		0.511			

^▲^T0-T2

^*^Mixed Model ANOVA

^a^*p*-value within groups

^b^*p*-value between groups

The effects of different tooth positions and tooth surfaces showed no statistically significant differences (*p* > 0.050) in the levels of plaque and the severity of gingivitis from baseline to three weeks after the intervention in the MTB and STB groups. Interestingly, the MCS group showed a statistically significant improvement in the severity of gingivitis of the anterior teeth compared to the posterior teeth [mean difference of 38.59 and 12.94, respectively, *p* = 0.037]. The MCS group also showed significant improvement in PISA values on the buccal surface compared to the palatal/lingual surface [mean difference of 40.03 and 11.63, respectively (*p* = 0.046)].

Meanwhile, the between-groups comparison effects of different tooth positions and tooth surfaces showed no statistically significant impact (*p* > 0.050) on the plaque levels. In terms of severity of gingivitis, the MCS group achieved a statistically significant improvement in the PISA values of the anterior teeth compared to the MTB and STB groups (*p* < 0.046 and *p* < 0.041, respectively). At the same time, the rest of the between-groups comparison showed no significant effect on the PISA values (*p* > 0.050).

## Discussion

According to the clinical results of this study, there was no significant difference between the three different types of oral hygiene tools in the reduction of the means of plaque scores and severity of gingivitis from baseline to three weeks following the intervention, suggesting that all three oral hygiene tools were equally effective in terms of good plaque control and gingival status. It thus has further highlighted that the outcomes of this trial were in accordance with the previously reported literature.

Research showed that *S. persica* chewing sticks were excellent anti-plaque and anti-gingivitis agents [19, 22]. An anti-plaque agent can reduce the plaque level to a threshold that exhibits benefits in preventing gingival inflammation and caries [45]. On the other hand, an anti-gingivitis agent is an oral hygiene apparatus that reduces gingival inflammation without necessarily impacting plaque levels [45]. The current findings confirm an earlier study that found both the *S. persica* chewing stick and the standard toothbrush are equally effective in reducing plaque [46] and gingival inflammation [47]. Following professional instruction on the proper use, studies reported that *S. persica* chewing sticks could be as effective as a toothbrush [22, 43] or even better in reducing plaque formation with a significant reduction of gingival inflammation and bleeding on probing [2, 48, 49]. Thus, as *S. persica* oral hygiene tools were shown to be good as a standard toothbrush in plaque and gingivitis control [15, 46, 50, 51], they can act as alternative oral hygiene armamentarium to those seeking oral and periodontal care with natural ingredients [35].

Several studies claimed the difficult handling of *S. persica* chewing stick due to the nature of its bristles which lie on the same long axis as the handle, may affect its therapeutic benefits in certain parts of the mouth [16, 49, 52]. Nonetheless, the present study reported no significant effects of plaque levels on different tooth positions and surfaces when comparing different oral hygiene tools. These findings were in accordance with the study by Batwa et al., despite their study population consisting of experienced miswak users [53]. Meanwhile, an earlier study stated that plaque reduction among miswak users was more significant in anterior than posterior teeth [22]. According to the authors, the study also involved habitual miswak users, who could remove plaque without being instructed on how to use the chewing stick most efficiently [22]. Hence, the findings reinforce the importance of correct techniques and appropriate oral hygiene advice to maintain good oral hygiene regardless of tooth locations and surfaces [2]. Moreover, patient-related factors also play an essential role in achieving effective toothbrushing [54]. Their good manual dexterity and positive behavioral aspects, such as high motivation and compliance shown among the participants of the present study, are the critical factors that influenced effective toothbrushing, regardless of the types of oral hygiene devices used [12].

In the present study, PISA values for the anterior teeth of the MCS group demonstrated a statistically significant difference compared to the posterior teeth. Interestingly, a statistically significant improvement in the severity of gingivitis of the anterior teeth was also obtained in the MCS group when compared with the MTB and STB groups. Although an earlier study justified that effective *S. persica* chewing sticks use was not limited to the anterior teeth, the users appeared to be more concerned about the anterior teeth and cleaning them more thoroughly than the posterior ones [50]. Easy access to the anterior segment has allowed its fibers to remove the plaque while simultaneously massaging the gingiva to safeguard against gingival inflammation [14]. Additionally, the significant improvement of PISA values of anterior teeth in the MCS group was noted as the *S. persica* chewing stick was used for a longer duration (5–10 min) than that of the *S. persica* and standard toothbrushes, hence might contribute to the favorable results [14]. In the MCS group, the PISA values for the buccal surface showed significant improvement in comparison to the palatal/lingual surface, which was in line with the previous studies [2, 22]. The nature of the miswak bristle on the long axis of the miswak has fashioned its easier access to the buccal surface compared to the palatal/lingual surfaces [13, 16]. Previous studies also reported that The reduced access to palatal and lingual surfaces might lead to gingival inflammation on these surfaces [22, 33], which was not reported in the present study. Moreover, the differences in PISA values between buccal and palatal/lingual tooth surfaces were insignificant when compared to the three groups.

This clinical trial presents an evident limitation of using a convenient sample population. Selection bias should be considered as highly driven participants with good oral hygiene status were more likely to participate. Age and gender were also not considered during randomization of the sample population. Hence, apart from being highly motivated and compliant, the ability to generalize the findings of this study is limited by the strict criteria of the participants involved, who were highly motivated and compliant. Nevertheless, efforts were made to minimize confounding factors among the participants. Participants with dental backgrounds, i.e., dental students, hygienists, and dentists, were excluded from the study to ensure the homogenous nature of all groups. The non-significant differences between the demographic characteristics and clinical parameters at baseline also supported the homogeneity among the participants at the beginning of the study.

Apart from that, the improvement in clinical parameter outcomes of the present study could be due to the well-recognized Hawthorne effect. The improvement in participants’ oral hygiene status as the result of their awareness of participating in this clinical trial, irrespective of the treatment modality [53]. Therefore, to overcome the influence of behavioral changes, Periodontal Inflamed Surface Area (PISA) was evaluated as an element in the gingival condition rather than taking the plaque index as the sole clinical parameter. PISA reflects the bleeding score of the gingiva and portrays the pharmacological response of the gingival tissue towards the efforts of oral hygiene care performed using the variety of oral hygiene tools [1, 12]. Thus, the consistency of reduction in both plaque level and severity of gingivitis reflected in the results could be an assurance factor for the preciseness of evaluation, serving as an effort to overcome the influence of the Hawthorne effect [55].

The current trial was not designed to assess the anticariogenic property of the oral hygiene tools, and therefore, cannot indicate effective equivalence between the *S. persica* chewing stick and the standard toothbrush pertinent to dental caries. The potential effects of confounding factors on gingival inflammation, such as dietary influence and lifestyle, were not addressed in the present study. Considering more communities are exposed to sedentary modern lifestyle and western diet that further evoke gingival inflammatory state [8, 56], it is hence essential to approach the interpretation of the findings with caution.

### Clinical implications and conclusion

On a short-term basis, *S. persica* chewing sticks, *S. persica* toothbrush, and the standard toothbrush are equally reliable in controlling plaque and gingivitis, provided that the cleaning is sufficiently thorough and performed regularly. A clear and specific protocol applied in this trial may promote the establishment of consistent guidelines for each type of oral hygiene device use, allowing for better uniformity in future trials and comparative effectiveness of similar oral hygiene products.

Incorporating *S. persica* into a design of a standard toothbrush merges the practicality of the standard toothbrush use with the bristles that possess the benefits of natural sources, representing a transitional step towards a modern-contemporary oral hygiene device. The promising short-term results in controlling plaque and gingival inflammation enable the practicality of the *S. persica* toothbrush use, for instance by the military personnel during their deployment in an isolated or confined environment. Despite the encouraging short-term results in controlling plaque and gingival inflammation, the current study could not conclusively affirm the effectiveness of this newly invented oral hygiene tool as an alternative in long-term use. Using the *S. persica* toothbrush as an effective oral hygiene tool should be further supported with scientific investigations in a more generalized cohorts with longer experiment times.

## Data Availability

The datasets used and analyzed during the current study and coding are available from the corresponding author on reasonable request.

## Abbreviations

MCS: Miswak chewing stick
MTB: Miswak toothbrush
STB: Standard toothbrush
PI: Plaque Index
PESA: Periodontal Attachment Surface Area
PISA: Periodontal Inflamed Surface Area
T0: Baseline
T1: One week post intervention
T2: Three weeks post intervention
